# Determinants of Outcome with Reoperative Surgery for Pseudomyxoma Peritonei in 186 Patients

**DOI:** 10.1097/AS9.0000000000000335

**Published:** 2023-09-15

**Authors:** Paul H. Sugarbaker, David Chang

**Affiliations:** From the ^*^Program in Peritoneal Surface Malignancy, Washington Cancer Institute, Washington, DC; † Westat, Rockville, MD.

**Keywords:** peritoneal metastases, cytoreductive surgery, low-grade appendiceal mucinous neoplasm, mucinous appendiceal adenocarcinoma

## Abstract

**Objective:**

To describe the long-term survival and clinical- and treatment-related variables that determine the outcome of repeat cytoreductive surgery (CRS) for mucinous appendiceal neoplasms with peritoneal dissemination.

**Summary Background:**

After patients with peritoneal dissemination of an appendiceal mucinous neoplasm have a CRS, disease progression may require secondary cytoreductive surgery (SCRS) and other treatments performed in a timely manner to prolong survival and help preserve an optimal quality of life.

**Methods:**

The clinical- and treatment-related variables associated with the index CRS and the SCRS were statistically assessed for their impact on survival.

**Results:**

One hundred eighty-six of 687 complete CRS patients (27.1%) had SCRS. The median follow-up was 10 years and the median survival was 12 years. There were 95 males (51%) and the median age was 45.0 years. Survival benefit was associated with the index CRS by use of early postoperative intraperitoneal chemotherapy (EPIC) with 5-fluorouracil [Hazard ratio (HR), 0.4; *P* = 0.0004]. Also, survival of low-grade mucinous appendiceal neoplasms versus mucinous appendiceal adenocarcinoma (HR, 2.8; *P* < 0.0001) was improved. The interval between index CRS and SCRS was significant at ≤12 months versus 12–36 months versus >36 months (*P* < 0.0001). Change in peritoneal cancer index and disease distribution as focal or diffuse was significant by univariant and multivariant analyses.

**Conclusions:**

If the CRS was complete, the use of EPIC 5-fluorouracil, the interval between the index CRS and the SCRS, the histologic grade of the mucinous neoplasm, and the extent of recurrent disease were prognostic variables that should be used to help select patients for SCRS.

## Abstract 2

Mini Abstract:

Appendiceal mucinous neoplasms are treated as a standard of care using cytoreductive surgery and hyperthermic intraperitoneal chemotherapy. A repeat cytoreductive surgery was performed in one quarter of our patients. The results of repeat surgery and the clinical- and treatment-related variables determined to be of benefit are presented.

## INTRODUCTION

Pseudomyxoma peritonei is defined as a spectrum of histologic subtypes of mucinous neoplasms emanating from the appendix and disseminating within the peritoneal spaces by redistribution.^1^ Management strategies have changed dramatically over the last 4 decades. This disease, when confined to the abdomen and pelvis, was treated in the past by repeated evacuation of mucinous tumors and surgical debulking with an inevitable lethal outcome.^2^ Currently, the treatment of moderate and large-volume disease is cytoreductive surgery (CRS) to remove all visible evidence of mucinous tumors followed by perioperative chemotherapy washing of the peritoneal cavity to eradicate microscopic disease.^3^ This potentially curative approach is now the standard of care.^4^ At the time of index cytoreduction, decisions regarding when to move ahead with extensive peritonectomy and visceral resections are usually not difficult. Total eradication of all disease with all histologic subtypes of the appendiceal mucinous neoplasm is the accepted goal of optimal treatment.^5^ The difficult judgment at this point in time regard is when not to operate.^6^ For example, early in the natural history of this disease, the perforated appendix may leak mucus or mucus plus epithelial cells into the right paracolic sulcus. The decision to operate and employ CRS and hyperthermic intraperitoneal chemotherapy (HIPEC), versus a watch-and-wait policy is not standardized.^7^ Another even more complex judgment regards when to and when not to reoperate after a definitive CRS plus perioperative chemotherapy has failed and the disease is recurrent. When is a second or sometimes a third or fourth surgery with an intent to cure or to maximally palliate the pseudomyxoma patient indicated.^8,9^

The goal of this article is to use a statistical analysis of clinical and histologic features obtained from index CRS and secondary cytoreductive surgery (SCRS) procedures to assist the surgeon in the decision-making process for SCRS. Of course, the condition of the patient (fitness for major surgery) and the patient’s symptoms must be factored into these recommendations.

## METHODS

### Patients

Prospectively recorded clinical information from a standardized database was retrospectively collected and statistically analyzed. All records were maintained in secured files. Data from referring institutions regarding the primary appendiceal neoplasm were stored in the same secured files. Consecutive patients from July 1982 to July 2020 are included. All patients had a diagnosis of perforated mucinous appendiceal neoplasm with peritoneal dissemination of mucus plus tumor cells or mucus only. Patients having a routine jejunostomy or ileostomy closure are not included in this group of patients. Also, patients having a reoperation for adhesions or a reoperation for another diagnosis such as lymphoma are not included unless mucinous appendiceal adenocarcinoma was confirmed and attempts at its resection performed. All patients had a complete or near complete cytoreduction scored as CC-0 or CC-1 at the index CRS.^10^ Before the initiation of this study, permission was obtained from the MedStar Georgetown Office of Research Integrity to analyze these retrospective data.

### Clinical- and Treatment-Related Variables Resulting from Index Cytoreductive Surgery

The age, gender, and interval from diagnosis of the primary appendiceal neoplasm until the performance of the index CRS was determined. Carcinoembryonic antigen (CEA) and cancer antigen 19-9 (CA19-9) were available for the majority of patients within 1 week before the index CRS. The prior surgical score was determined from a review of operative notes and pathology reports from referring institutions.^10^ A score of 0, 1, and 2 was compared with a score of 3. The peritoneal cancer index (PCI) at the time of the index CRS was analyzed as low,^3–14^ moderate,^15–29^ or high (>30).^10^

A number of treatment-related variables were recorded from the index CRS. Time required for CRS, the number of visceral resections performed, presence vs. absence of total colectomy, presence versus absence of total or near total gastrectomy, and presence versus absence of right colon resection were statistically analyzed for their impact on survival after SCRS. Ostomy construction was recorded as performed or absent at the time of the index CRS.

The impact of perioperative chemotherapy on subsequent SCRS was determined by comparing no perioperative chemotherapy to early postoperative intraperitoneal chemotherapy (EPIC mitomycin C plus EPIC 5-fluorouracil), to HIPEC mitomycin C plus EPIC 5-fluorouracil, and to HIPEC mitomycin C only.^11^ The goal of this assessment was to determine the possible impact of EPIC and/or HIPEC on subsequent SCRS. The histology was assessed from multiple specimens separately submitted and individually processed, with several slides read from each specimen. The histologic subtypes of these mucinous appendiceal neoplasms followed the description of Ronnett et al^12^ The acronyms were taken from the publications of Misdraji et al^13^ The low-grade appendiceal mucinous neoplasms (LAMN) were composed of mucus containing variable amounts of mildly atypical cells. The second low-grade subtype was the mucinous appendiceal adenocarcinoma of intermediate type (MACA-Int).^12^ This tumor was largely LAMN histology with foci of mucinous adenocarcinoma in less than 5% of the field of view. Mucinous appendiceal adenocarcinoma grade 1 (MACA-1) showed minimal atypia but was invasive in the peritoneum. It was well differentiated. MACA grade 2 (MACA-2) was moderately differentiated and MACA grade 3 (MACA-3) was poorly differentiated. If signet ring cells were present the histologic subtype was designated MACA-S.^14^ Because MACA-1, 2, 3, and S have been shown to have very similar survival when treated by CRS and HIPEC, they were included together for the survival analysis in Table 1 as MACA.^15^ If lymph nodes were found to be positive when right colectomy or CRS were performed, the histology was designated as MACA lymph node positive (MACA-LN).^16^

**TABLE 1.** Univariant Statistical Analysis of Clinical- and Treatment-Related Variables as a Result of the Index Cytoreductive Surgery in 186 Patients on the Outcome of Secondary Cytoreductive Surgery for Low-Grade Appendiceal Mucinous Neoplasm and Mucinous Appendiceal Adenocarcinoma

Variable With CRS	No. (%)	Median Survival (Years)	Hazard Ratio (95% CI)	*P* Value
Age at time of CRS				
18–45 >45	94 (51%) 92 (49%)	12.0 11.0	Reference 1.0 (0.68, 1.40)	0.8932
Gender				
Male Female	95 (51%) 91 (49%)	12.0 12.0	Reference 1.0 (0.73, 1.49)	0.8198
Interval from diagnosis to CRS				
0–12 months >12 months	130 (70%) 56 (30%)	14.0 9.0	Reference 1.5 (0.99, 2.11)	0.0549
Carcinoembryonic antigen (µg/ml) (N = 160)				
<10 10–50 >50	69 (43%) 55 (34%) 36 (23%)	12.0 12.0 9.0	Reference 0.9 (0.60, 1.49) 1.3 (0.82, 2.18)	0.7936 0.2385
Cancer antigen 19–9 (units/ml) (N = 145)				
<50 50–1000 >1000	64 (44%) 62 (43%) 19 (13%)	14.0 11.0 12.0	Reference 1.4 (0.88, 2.14) 1.3 (0.70, 2.42)	0.1626 0.4025
Prior surgical score				
0, 1, or 2 3	133 (72%) 53 (28%)	12.0 10.0	Reference 1.2 (0.85, 1.84)	0.2597
Peritoneal cancer index				
3–14 (low) 15–30 (moderate) >30 (high)	29 (16%) 102 (55%) 55 (30%)	7.0 13.0 12.0	Reference 0.6 (0.38, 0.99) 0.7 (0.38, 1.12)	0.0487 0.1230
Time for CRS				
1–11 hours >11 hours	101 (55%) 81 (45%)	11.0 12.0	Reference 0.9 (0.63, 1.31)	0.5974
Visceral resections				
None 1 2 3 or more	38 (20%) 48 (26%) 54 (29%) 46 (25%)	12.0 17.0 11.0 11.0	Reference 0.8 (0.48, 1.45) 1.1 (0.66, 1.88) 1.1 (0.67, 1.95)	0.5252 0.6994 0.6199
Total colectomy				
No Yes	156 (84%) 30 (16%)	13.0 8.0	Reference 1.5 (0.94, 2.39)	0.0929
Total gastrectomy				
No Yes	140 (75%) 46 (25%)	14.0 8.8	Reference 1.6 (1.07, 2.31)	0.0221
Right colon resection				
No Yes	98 (53%) 88 (47%)	15.0 9.0	Reference 1.4 (0.97, 2.01)	0.0694
Ostomy construction				
No Yes	131 (70%) 55 (30%)	14.0 9.0	Reference 1.4 (0.96, 2.07)	0.0799
Perioperative chemotherapy with index CRS				
None EPIC mitomycin C+EPIC 5-fluorouracil HIPEC plus EPIC 5-fluorouracil HIPEC only	6 (3%) 49 (26%) 40 (22%) 90 (49%)	4.3 14.0 21.0 8.0	2.6 (1.11, 6.04) 0.6 (0.40, 0.97) 0.4 (0.22, 0.64) Reference	0.0275 0.0360 0.0004
Histopathology from right colon resection and index CRS				
LAMN MACA-Intermediate MACA MACA with positive lymph nodes	88 (48%) 17 (9%) 67 (36%) 12 (7%)	15.3 15.0 6.0 4.5	Reference 1.1 (0.55, 2.30) 2.8 (1.86, 4.21) 3.9 (1.99, 7.61)	0.0984 <0.0001 <0.0001

CI indicates confidence interval; CRS, cytoreductive surgery; EPIC, early postoperative intraperitoneal chemotherapy; HIPEC, hyperthermic intraperitoneal chemotherapy; LAMN, low-grade appendiceal mucinous neoplasm; MACA, mucinous appendiceal adenocarcinoma.

A univariant and a multivariant analysis of these variables are presented in Tables 1 and 2, respectively.

**TABLE 2.** Multivariant Analysis of Clinical- and Treatment-Related Variables as a Result of the Index Cytoreductive Surgery in 186 Patients on the Outcome of Secondary Cytoreductive Surgery for Low-Grade Appendiceal Mucinous Neoplasm and Mucinous Appendiceal Adenocarcinoma

Variable With CRS	No. (%)	Median Survival (Years)	Hazard Ratio (95% CI)	*P* Value
Total gastrectomy				
No Yes	140 (75%) 46 (25%)	14.0 8.8	Reference 1.5 (1.03, 2.30)	0.0377
Perioperative chemotherapy with index CRS				
None EPIC mitomycin C plus EPIC 5-FU HIPEC mitomycin C plus EPIC 5-FU HIPEC only	6 (3%) 49 (26%) 40 (22%) 90 (49%)	4.3 14.0 21.0 8.0	1.6 (0.65, 3.73) 0.6 (0.37, 0.95) 0.4 (0.23, 0.80) Reference	0.3257 0.0280 0.0075
Histopathology from right colon resection and index CRS				
LAMN MACA-Intermediate MACA MACA with positive lymph nodes	88 (48%) 17 (9%) 67 (36%) 12 (7%)	15.3 15.0 6.0 4.5	Reference 0.6 (0.30, 1.40) 2.8 (1.22, 3.84) 3.8 (1.87, 7.50)	0.2668 0.0176 0.0002

5-FU indicates 5-fluorouracil CI, confidence interval; CRS, cytoreductive surgery; EPIC, early postoperative intraperitoneal chemotherapy; HIPEC, hyperthermic intraperitoneal chemotherapy; LAMN, low-grade appendiceal mucinous neoplasm; MACA, mucinous appendiceal adenocarcinoma.

### Clinical- and Treatment-Related Variables as a Result of the Secondary Cytoreductive Surgery

The symptoms or signs recorded in an interview with the patient before SCRS were prospectively recorded. Patients were assessed as asymptomatic, progressing in follow-up by CT, CEA, or CA19-9 tumor markers, presenting with a mass in the abdomen, pelvis, or abdominal wall, ascites, bowel obstruction, or other symptoms or signs. The most predominant symptom was recorded. Patients were also assessed as no symptoms present vs. symptomatic. The interval between CRS and SCRS was determined. It was statistically analyzed as ≤12 months, 12–36 months, and >36 months.

The PCI was determined at the time of SCRS.^10^ It was analyzed as low,^3–14^ moderate,^15–29^ or high (>30). A change in the PCI as compared with that recorded at the time of the original CRS was analyzed.^17^ If the PCI at the SCRS was less than half of the PCI at the index CRS, the change in the PCI was recorded as less. If they were approximately the same, this was recorded. If the PCI had increased from the time of the index CRS to the SCRS the change in PCI was recorded as increased. Completeness of cytoreduction score was available and statistically analyzed as CC-0 and CC-1 versus CC-2 versus CC-3.^10^ Technical features related to the SCRS regarding the time required for the reoperative event, ostomy construction versus no construction, visceral resections none versus 1 versus 2 or 3, presence versus absence of total colectomy, total or near total gastrectomy, and right colon resection were recorded. The disease distribution within the peritoneal spaces was estimated as focal versus diffuse. Focal disease was limited to 2 abdominal-pelvic regions, and diffuse disease was documented in 3 or more regions.^10^

Patients who had perioperative chemotherapy as a part of the SCRS were compared with those who did not have additional perioperative chemotherapy. Perioperative chemotherapy was recorded as EPIC, HIPEC plus EPIC, or HIPEC only. If a change in the HIPEC occurred, this was noted and analyzed for its impact on survival. Five different perioperative chemotherapy regimens were employed as the institutional review board-approved pharmacologic studies were completed and the standardized orders were approved by the pharmacy committee. These regimens were: (1) EPIC mitomycin C plus 5-fluorouracil or no HIPEC, (2) HIPEC mitomycin C, (3) HIPEC cisplatin plus doxorubicin, (4) HIPEC melphalan, or (5) HIPEC mitomycin C plus doxorubicin.^11^ Regimen 1 was SCRS in the absence of HIPEC. Regimens 2 through 5 included hyperthermia with perioperative chemotherapy. Another assessment of perioperative hyperthermic intraperitoneal chemotherapy compared HIPEC mitomycin C to HIPEC cisplatin/doxorubicin, to HIPEC melphalan, to HIPEC mitomycin C/doxorubicin. Only patients who received HIPEC as a part of the SCRS were included in this type of HIPEC analysis.

### Morbidity and Mortality

The morbidity and mortality over the long time span of this study were determined by a prospective evaluation of adverse events.^18,19^ Class 1, 2, and 3 adverse events are not tabulated in this article. Class 4 adverse events required return to the operating theater and class 5 indicated postoperative death.

A univariant and a multivariant analysis of variables associated with SCRS is presented in Tables 3 and 4.

**TABLE 3.** Univariant Statistical Analysis of Clinical- and Treatment-Related Variables as a Result of a Secondary Cytoreductive Surgery in Patients with Low-Grade Appendiceal Mucinous Neoplasm and Mucinous Appendiceal Adenocarcinoma

Variable with SCRS	No. (%)	Median Survival (years)	Hazard Ratio (95% CI)	*P* Value
Indications for SCRS				
Asymptomatic for second-look after NAC	28 (15.1%)	7.0	Reference	
Progression in follow-up by CT/tumor				
markers Mass in abdomen or pelvis Ascites Bowel obstruction Other	98 (52.7%) 16 (8.6%) 9 (4.8%) 28 (15.1%) 7 (3.8%)	14.0 NA 10.0 4.0 12.0	0.6 (0.34, 0.93) 0.3 (0.11, 0.74) 0.9 (0.36, 2.21) 1.6 (0.88, 2.78) 0.5 (0.18, 1.55)	0.0238 0.0105 0.8039 0.1299 0.2444
Symptoms				
No symptoms present Symptomatic	126 (67.7%) 60 (32.3%)	13.0 8.5	Reference 1.3 (0.90, 1.92)	0.1553
Interval from index CRS to SCRS				
<12 months 12–36 months >36 months	37 (20%) 84 (45%) 65 (35%)	5.0 7.0 23.0	3.3 (1.93, 5.47) 2.4 (1.57, 3.78) Reference	<0.0001 <0.0001
Peritoneal cancer index at SCRS				
0–14 (low) 15–30 (moderate) >30 (high)	114 (64%) 63 (35%) 2 (1%)	15.0 7.0 NR	Reference 1.9 (1.29, 2.71) 2.0 (0.27, 14.3)	0.0010 0.5003
Change in peritoneal cancer index				
Less Same Increased	108 (58%) 55 (30%) 22 (12%)	16.0 5.0 7.0	Reference 2.1 (1.43, 3.21) 2.4 (1.40, 3.96)	0.0002 0.0013
Completeness of cytoreduction at SCRS				
CC-0 and CC-1 CC-2 CC-3	123 (71%) 18 (10%) 33 (19%)	17.0 10.0 4.0	Reference 1.7 (0.93, 3.06) 3.6 (2.31, 5.75)	0.0855 <0.0001
Time of SCRS				
1–8 hours >8 hours	128 (73%) 47 (27%)	14.0 7.0	Reference 1.5 (1.02, 2.27)	0.0398
Ostomy construction				
No Yes	153 (82%) 33 (18%)	14.0 7.0	Reference 2.0 (1.29, 0.79)	0.0012
Visceral resection				
None 1 2 or 3	98 (53%) 50 (27%) 38 (20%)	12.0 14.0 7.0	Reference 0.9 (0.56, 1.36) 1.2 (0.76, 1.88)	0.5471 0.4327
Total colectomy				
No Yes	172 (93%) 14 (7%)	12.0 6.0	Reference 1.5 (0.81, 2.82)	0.1917
Gastrectomy				
No Yes	175 (94%) 11 (6%)	12.0 7.0	Reference 1.1 (0.56, 2.34)	0.7224
Right colon resection				
No Yes	152 (82%) 34 (18%)	12.0 7.0	Reference 1.5 (0.94, 2.27)	0.0959
Disease distribution within peritoneal spaces				
Focal Diffuse	118 (63%) 68 (37%)	NR 7.0	Reference 2.8 (1.80, 4.22)	<0.0001
Perioperative chemotherapy with SCRS				
Yes No	113 (61%) 73 (39%)	12.0 12.0	Reference 1.0 (0.67, 1.40)	0.8492
Change in HIPEC CRS to SCRS				
Yes No	76 (41%) 110 (59%)	11.0 12.0	Reference 1.0 (0.68, 1.42)	0.9276
Perioperative chemotherapy with SCRS				
EPIC or no HIPEC HIPEC mitomycin C HIPEC cisplatin/doxorubicin HIPEC melphalan HIPEC mitomycin C/doxorubicin	95 (51.1%) 46 (50.5%) 19 (20.9%) 19 (20.9%) 7 (7.7%)	12.0 8.5 5.0 16.0 17.0	Reference 1.1 (0.69, 1.62) 1.6 (0.91, 2.83) 0.6 (0.27, 1.30) 0.7 (0.26, 1.98)	0.8057 0.1025 0.1908 0.5259
HIPEC regimen (N = 91 HIPEC patients only)				
HIPEC mitomycin C HIPEC cisplatin/doxorubicin HIPEC melphalan HIPEC mitomycin C/doxorubicin	46 (50.5%) 19 (20.9%) 19 (20.9%) 7 (7.7%)	8.5 5.0 16.0 17.0	1.8 (0.78, 4.05) 2.7 (1.10, 6.66) Reference 1.2 (0.36, 4.16)	0.1676 0.0299 0.7543
Class 4 adverse events (N = 185)				
Present None	12 (7%) 173 (93%)	6.0 12.0	1.9 (0.99, 3.64) Reference	0.0530

CI indicates confidence interval; CRS, cytoreductive surgery; EPIC, early postoperative intraperitoneal chemotherapy; HIPEC, hyperthermic intraperitoneal chemotherapy; NAC, neoadjuvant chemotherapy; NR, median survival not reached; SCRS, secondary cytoreductive surgery.

**TABLE 4.** Multivariant Analysis of Clinical- and Treatment-Related Variables as a Result of a Secondary Cytoreductive Surgery in Patients with Low-Grade Appendiceal Mucinous Neoplasm and Mucinous Appendiceal Adenocarcinoma

Variable With SCRS	No. (%)	Median Survival (years)	Hazard Ratio (95% CI)	*P* Value
Interval from index CRS to SCRS				
<12 months 12–36 months	37 (20%) 84 (45%)	5.0 7.0	4.3 (2.46, 7.65) 2.3 (1.46, 3.73)	<0.0001 0.0004
>36 months	65 (35%)	23.0	Reference	
Change in peritoneal cancer index				
Less Same Increased	108 (58%) 55 (30%) 22 (12%)	16.0 5.0 7.0	Reference 2.2 (1.40, 3.42) 2.5 (1.43, 4.46)	0.0006 0.0014
Disease distribution within peritoneal spaces				
Focal Diffuse	118 (63%) 68 (37%)	NR 7.0	Reference 2.5 (1.52, 4.02)	0.0003
HIPEC regimen (all patients)				
None + EPIC HIPEC mitomycin C HIPEC cisplatin/doxorubicin HIPEC melphalan HIPEC mitomycin C/doxorubicin	95 (51%) 46 (25%) 19 (10%) 19 (10%) 7 (4%)	12.0 8.5 5.0 16.0 17.0	3.9 (1.72, 8.76) 2.0 (0.88, 4.70) 2.1 (0.87, 5.29) Reference 1.6 (0.47, 5.61)	0.0011 0.0970 0.0993 0.4467

CI indicates confidence interval; CRS, cytoreductive surgery; EPIC, early postoperative intraperitoneal chemotherapy; HIPEC, hyperthermic intraperitoneal chemotherapy; NR, median survival not reached; SCRS, secondary cytoreductive surgery.

### Statistical Analysis

These data were processed using R 4.0.0, R Foundation for Statistical Analysis, Vienna, Austria. The Kaplan–Meier method estimated overall survival. The *P* value was set at <0.05. Multivariant analysis was performed by the Cox stepwise regression model. The start time for all analyses was the date of CRS, and the stop date was overall survival.

In Table 5, selected variables associated with the 3 major histologic subtypes of pseudomyxoma peritonei are presented. These subtypes are LAMN, MACA, and MACA-LN. The table provides the percent of each subgroup that underwent SCRS, and the statistical significance of a particular variable within a particular histologic subtype.

**TABLE 5.** Comparisons of Selected Variables Associated With Three Histologic Subtypes When a Second Cytoreductive Surgery is Performed

	LAMN	MACA	MACA-LN
Number of patients with complete index CRS	450	198	39
Number of patients having SCRS	88	86	12
% of subgroup having SCRS	19.6	43.4	30.8
Median survival	15.3 years	6.0 years	4.5 years
Significance of index CRS to SCRS interval	*P* = 0.0013	*P* = 0.0059	*P* = 0.1453
Significance of complete CRS with SCRS	*P* = 0.0104	*P* = 0.0014	*P* = 0.1356
Significance of EPIC 5-fluorouracil at index CRS on SCRS survival	*P* = 0.0095	*P* = 0.5053	*P* = 0.0469
Disease recurrence described as focal versus diffuse	*P* = 0.0001	*P* = 0.0369	*P* = 0.0526

CRS indicates cytoreductive surgery; LAMN, low-grade appendiceal mucinous neoplasm; MACA, mucinous appendiceal adenocarcinoma; MACA-LN, mucinous appendiceal adenocarcinoma lymph node, SCRS, secondary cytoreductive surgery.

## RESULTS

### Univariant Analysis of Clinical- and Treatment-Related Variables as a Result of Index CRS

The appendiceal peritoneal metastases database identified 687 patients who had a CC-0/1 index CRS. A selected group of these patients (186, 26.7%) had a complete cytoreductive surgery, LAMN, or MACA histopathology of the peritoneal metastases, an absence of disease outside of the peritoneal spaces, and an SCRS with histologic confirmation of recurrent disease. The first patient to receive these treatments underwent cytoreductive surgery in 1982. The most recent cytoreduction was in 2020. Univariant statistical analysis of clinical- and treatment-related variables as a result of the index CRS in patients having an SCRS is shown in Table 1. The age and gender were not significant. The interval from diagnosis to CRS was of borderline significance in favor of a 1-year interval as compared with a many-year interval [Hazard ratio (HR), 1.5; *P* = 0.0549]. The level of tumor markers, prior surgical score, PCI, hours required for the index CRS, number of visceral resections, and performance of total colectomy were not significant. The need for total gastrectomy at the time of index CRS did adversely affect the outcome of SCRS (HR, 1.6; *P* = 0.0221). Right colon resection and ostomy construction were not significant. The use of HIPEC mitomycin C plus EPIC 5-fluorouracil at the time of index CRS did cause a marked improvement in survival as compared with HIPEC only (HR, 0.4; *P* = 0.0004). Also, EPIC mitomycin C plus EPIC 5-fluorouracil was statistically different from HIPEC only (HR, 0.6; *P* = 0.0360). The data suggests that EPIC 5-fluorouracil caused superior survival when compared with HIPEC only.

The histologic subtype determined from the peritoneal metastases at the index CRS was not significant when LAMN was compared with MACA-Int. However, when compared with MACA (HR, 2.8; *P <* 0.0001) and MACA-LN (HR, 3.9; *P <* 0.0001), the histologic subtype was very significant.

### Multivariant Analysis of Clinical- and Treatment-Related Variables of Index CRS

The multivariant analysis of clinical- and treatment-related variables from the index CRS are displayed in Table 2. The large number of patients (25%) requiring a total gastrectomy with index CRS in our attempt to achieve a complete index CRS allowed this extensive procedure to be associated with reduced survival (HR, 1.5; *P* = 0.0377). Other extended visceral resections, such as total colectomy, right colon resection, or ostomy construction, did not reduce survival in the multivariant analysis.

The use of EPIC 5-fluorouracil lavage of the abdominal and pelvic space was associated with improved survival. EPIC mitomycin C plus EPIC 5-fluorouracil (HR, 0.6; *P* = 0.0280) or HIPEC mitomycin C plus EPIC 5-fluorouracil (HR, 0.4; *P* = 0.0075) at the time of index CRS caused improved survival with the SCRS. This statistic supports the consistent observation at the time of SCRS that the intestinal adhesions are greatly reduced in the patients who had EPIC 5-fluorouracil as part of the index CRS.

Patients with the closely related histologic subtypes of LAMN and MACA-Int did not show a survival difference when subjected to SCRS. However, when the MACA (HR, 2.8; *P* = 0.0176) or MACA-LN (HR, 3.8; *P* = 0.0002) histologic subtype was compared with the LAMN subtype, there was a marked difference in survival. The median survival decreased from 15.3 years to 6.0 and 4.5 years, respectively.

### Univariant Analysis of Clinical- and Treatment-Related Variables of SCRS

Univariant statistical analysis of clinical- and treatment-related variables as a result of the SCRS is shown in Table 3. Patients’ symptoms at the time of SCRS showed marked differences in outcome. The poorest survival was in asymptomatic patients having SCRS as a second look after systemic chemotherapy treatment (median survival 7.0 years). Also, patients with bowel obstruction had a poor outcome with a median survival of 4 years. The best outcome was in those patients who had SCRS with recurrence detected in follow-up by CT or tumor markers CEA or CA19-9 (HR, 0.6; *P* = 0.0238). Also, a mass in the abdomen or pelvis was associated with improved survival (HR, 0.2; *P* = 0.0105). Patients without symptoms compared with SCRS patients with symptoms did not show a statistically significant difference in survival.

As compared with a surgical interval of >36 months, a short interval between CRS and SCRS of ≤12 months was associated with the shortest survival of 5.0 years (HR, 3.3; *P* ≤ 0.0001). Comparison to an interval of 12–36 months (HR, 2.4; *P <* 0.0001) and an interval of >36 months (HR, 3.3; *P <* 0.0001) also showed marked differences.

The PCI, change in PCI, and completeness of cytoreduction score all showed significant differences. Also, variables that indicated an SCRS procedure was of greater magnitude, such as time for surgery of greater than 8 hours and ostomy construction, showed significantly reduced survival. An increased number of visceral resections, total colectomy, gastrectomy, and right colon resection, when required for SCRS were not significant. The disease distribution determined by the surgeon as diffuse involving more than 2 abdominal-pelvic regions was associated with reduced survival as compared with focal disease (HR, 2.8; *P <* 0.0001).

Patients who had perioperative chemotherapy with HIPEC or HIPEC plus EPIC had a 12-year median survival. If no perioperative chemotherapy was used, the median survival was also 12 years. A change in perioperative chemotherapy between index CRS and SCRS had no impact on survival. Also, when no hyperthermic perioperative chemotherapy was compared with 4 different HIPEC regimens there was no significant difference.

In an effort to identify a HIPEC regimen of benefit in SCRS patients, an analysis of 91 patients who had HIPEC was performed. HIPEC mitomycin C, HIPEC cisplatin/doxorubicin, HIPEC melphalan, and HIPEC mitomycin C/doxorubicin were compared. HIPEC cisplatin/doxorubicin showed poor survival (HR, 2.8; *P* = 0.0238).

The occurrence of class 4 adverse events caused a borderline decreased in survival.

### Multivariant Analysis of Clinical- and Treatment-Related Variables of SCRS

The multivariant analysis of clinical- and treatment-related variables as a result of SCRS is displayed in Table 4. The interval from index CRS to SCRS had a large impact on survival. An interval of greater than 36 months showed a survival of 23 years as compared with 7.0 years for 12–36 months (HR, 2.3; *P* = 0.0004) and 5.0 years for less than 12 months (HR, 4.3; *P <* 0.0001). A change in the PCI between the time of index CRS and SCRS was significant. The same PCI or an increased PCI indicated a significantly reduced survival. If the disease distribution was limited to 2 abdominal-pelvic regions (focal disease), the survival was improved as compared with 3 or more abdominal-pelvic regions (diffuse disease) (HR, 2.5; *P* = 0.0003). In nonrandomized data regarding perioperative chemotherapy with SCRS, HIPEC with melphalan may have caused an improved survival when compared with patients who had no perioperative chemotherapy or EPIC.

### Morbidity and Mortality

There were no postoperative deaths in this group of patients. Two patients had leakage from the duodenal stump following gastrectomy. Five patients developed a small bowel fistula following extensive lysis of adhesions. Twelve patients required a return to the operating room for an incidence of grade 4 adverse events of 7.0%.

Table 5 compares selected variables associated with the 3 histologic subtypes of pseudomyxoma peritonei. The percent of patients with LAMN having SCRS was 19.6%, with MACA 43.4%, and with MACA-LN 30.8%. The surgical interval was significant with LAMN and MACA but not with MACA-LN. Also, the CC score with SCRS was significant for LAMN and MACA but not for MACA-LN. The improved survival when EPIC 5-fluorouracil was used with index CRS was significant with all 3 histologic subtypes. Disease recurrence described as focal versus diffuse was significant for all 3 histologic subtypes.

## DISCUSSION

### Search for Prognostic Indicators From Prior Publications

The strategy for a definitive management plan for the initial treatment of pseudomyxoma peritonei is well established.^4^ However, much has been written and many diverse opinions voiced regarding the selection of patients and a management plan for pseudomyxoma peritonei patients with recurrent disease. Yan et al^20^ reported on 111 patients with disease progression following CRS and perioperative intraperitoneal chemotherapy. Ninety-eight patients (88.2%) underwent a second surgery and 26 patients (23.4%) a third procedure. Focal versus diffuse disease and complete cytoreduction at the time of the second procedure were the significant risk factors determining survival. The most common sites for treatment failure after the first CRS and perioperative intraperitoneal chemotherapy were the small bowel, small bowel mesentery, and pelvis. The Kaplan–Meier survival estimated that 53 of the 98 reoperated patients (54.1%) were 10-year survivors.

Brouquet et al^21^ from the Gustave Roussy Cancer Institute used a limited extent of peritoneal disease and a disease-free interval of >12 months to select 12 patients with pseudomyxoma peritonei for a second procedure. The 5-year disease-free survival rate was 19%. This group suggests that a change in the perioperative chemotherapy regimen from the first to the second CRS may contribute to a favorable outcome.

Sardi et al^22^ reported on 162 pseudomyxoma peritonei patients, of whom 26 (16.0%) had a second CRS plus HIPEC. The mean disease-free interval between the first and second CRS was 23.1 months. Overall survival at 3 years was 54.3% and at 5 years was 33.9%. Histology was an important determinant of survival with 10 of 10 LAMN patients alive at 5 years after the SCRS and HIPEC and 0% at 5 years for MACA (*P* = 0.002).

Ahmadi et al^23^ from Basingstoke, UK, reported on 430 patients (37.6%) of 1145 pseudomyxoma peritonei patients treated by CRS and HIPEC who developed recurrent disease. Of these 430 patients with recurrence, 145 (33.7%) underwent repeat CRS, 119 (27.7%) had a watch-and-wait approach, and 119 (27.7%) had palliative chemotherapy. For patients who had a repeat CRS, prognostic factors for decreased survival in the multivariant analysis were early recurrence ≤1 year (*P* = 0.003), incomplete cytoreduction (*P* = 0.010), grade 3 complication (*P* = 0.32), and high-grade tumor (*P* = 0.019).

### Outcome of SCRS Dependent on Complete CRS

From the data in our current article regarding SCRS for appendiceal mucinous neoplasms, a favorable outcome is largely dependent on the completeness of cytoreduction, the histologic subtype of the peritoneal metastases, and the interval between CRS and SCRS. Our data and the literature review suggest that an elective SCRS is not indicated in patients whose preoperative workup does not indicate that a complete cytoreduction will occur. In these patients who have had extensive prior CRS, preoperative laparoscopy is not often feasible to assess the extent and location of the disease. Also, the preoperative radiologic workup is made difficult by the anatomic distortion and extensive scar tissue caused by extensive prior CRS. In our patients, those with the best prognosis were those in regular follow-up with repeated CT and serial tumor markers. A meticulous follow-up to achieve a serial clinical and radiologic assessment seemed to be of great value in this group of patients. However, the selection of patients who have a high likelihood of complete cytoreduction remains an unsolved problem. The lack of preoperative laparoscopic and reliable radiologic information causes the clinical- and treatment-related variables to be of considerable clinical value for selecting SCRS as a treatment option.

### Outcome of SCRS Dependent on Histologic Subtype

Our data places the histologic subtype as one of the most important determinants of outcome with SCRS. Overall median survival of LAMN patients with SCRS is 15.3 years, with MACA-1, 2, 3, and S 6.0 years, and with MACA-LN 4.5 years. These results are highly statistically significant by univariant and multivariant statistical analysis. Although the survival of MACA-LN patients in the absence of SCRS is superior to the survival of patients with recurrence requiring SCRS, the difference is not robust and only becomes apparent after 5 years of follow-up.^24^ The MACA-LN patients with a complete index CRS should have careful follow-up and be considered for SCRS only if a complete resection is predicted.

### Outcome of SCRS Dependent on Time Interval Between CRS and SCRS

Our data shows that a delay in index CRS in patients with mucinous appendiceal neoplasms detracts from long-term survival. If the interval from diagnosis to CRS was more than 1 year, median survival was 9.0 years. If 1 year or less, median survival was 14.0 years (*P* = 0.0549). In marked contrast, a long interval between the index CRS and the SCRS resulted in an improved outcome. An interval of less than 12 months, 12–36 months, and greater than 36 months showed median survivals of 5, 7, and 23 years (*P* ≤ 0.0001). Votanopoulos et al^25^ in a study of 62 patients with a variety of diagnoses who underwent SCRS, published that a statistically significant survival advantage was dependent on the interval between CRS and SCRS (*P* = 0.009). These authors stated that a long interval between the surgeries indicated a favorable biology of the tumor.^25^

### Outcome of SCRS Dependent on Patient Symptoms or Signs at the Time of SCRS

The status of the patient before SCRS and the indications for the repeat interventions were important determinants of the outcome. Fifteen percent were asymptomatic before the SCRS, having received one or more neoadjuvant chemotherapy regimens before a scheduled return to the operating room for a second-look surgery. Fifteen of these 28 patients had an ostomy closure as part of the second-look procedure. This group of patients had a median survival of 7 years. These patients, along with those who presented with bowel obstruction, had the poorest outcome of SCRS. The most favorable outcome was seen in those patients who, in follow-up, showed a gradual progression of disease detected by serial radiologic and/or tumor marker assessments. This indication for SCRS was seen in 52.7% of patients and was associated with a median survival of 14.0 years.

### Perioperative Chemotherapy with 5-Fluorouracil at the Time of CRS Improves the Outcome of SCRS

A review of the literature and our own extensive data regarding the impact of EPIC 5-fluorouracil on survival with index CRS showed no significant benefit from this treatment.^26^ However, our current data shows that the use of EPIC 5-fluorouracil with the index CRS may result in a significant improvement in survival with the SCRS. If EPIC mitomycin C plus EPIC 5-fluorouracil was used, the mean survival after SCRS was 14.0 years (*P* = 0.0360). If HIPEC plus EPIC 5-fluorouracil was used, the median survival was 21.0 years (*P* = 0.0004). If HIPEC only was used, the median survival dropped to 8.0 years. The use of EPIC 5-fluorouracil with the index CRS seemed to facilitate the beneficial effects achieved with SCRS. There may be an important explanation for the improved survival shown for EPIC 5-fluorouracil with index CRS when SCRS is required. Prior publications show that EPIC 5-fluorouracil diminishes the adhesive process associated with CRS plus HIPEC. Thereby the use of EPIC 5-fluorouracil allowed for a more complete abdominal exploration for removal of small-volume residual disease as a result of less scar tissue apparent at the SCRS.^27,28^ This observation regarding the delayed benefit of EPIC 5-fluorouracil needs to be further investigated with propensity score matching and temporal analysis of trends before application to clinical practice. It is presented as a preliminary observation in need of confirmation.

### Comparison of Selected Variables Associated With Three Histologic Subtypes When SCRS is Performed

From the data presented in this article and from prior publications on SCRS^24,29,30^ it is possible to find some meaningful comparisons that LAMN histology, MACA histology, and MACA-LN histology display. As shown in Table 5, of the 186 SCRS procedures, 88 of 450 (19.6%) of the LAMN subgroup had SCRS, 86 of 198 (43.4%) of the MACA subgroup had SCRS, and 12 of 39 (30.8%) of LAMN-LN had SCRS. If EPIC 5-fluorouracil with the index CRS does improve the outcome of SCRS as suggested by Tables 1 and 2, the large percentage of MACA patients having SCRS makes the use of EPIC 5-fluorouracil reasonable in this group of patients. Table 5 shows the marked difference in median survival with SCRS of LAMN, MACA, and MACA-LN histologic subtypes.^24,29,30^ The significance of CRS to SCRS interval on survival is variable between the histologic subtypes, as is the significance of complete CRS and the impact of EPIC 5-fluorouracil with index CRS on SCRS survival. The simple observation of focal versus diffuse disease remains a significant determinant of survival for all 3 histologic subtypes.^24,29,30^

### Perioperative Intraperitoneal Chemotherapy With SCRS Did Not Improve Survival

If one reviews the pharmacology of intraperitoneal chemotherapy administration, it becomes clear that the penetration of the drugs into peritoneal metastases and into the normal peritoneum is limited.^11^ The intraperitoneal chemotherapy moves into the surfaces of the abdomen and pelvis by simple diffusion. This means that the concentration difference of chemotherapy within the peritoneal space and within the tissues that are immediately adjacent drives the movement of cancer chemotherapy into the tissues. One of the most obvious differences in the SCRS procedure as compared with an index CRS procedure is the extent of the adhesions which must be divided before the resection of recurrent disease can occur. These adhesions are, as much as possible, resected rather than merely divided. However, the extent of scar tissue on abdominal, pelvic, and visceral surfaces may severely impair the efficacy of perioperative intraperitoneal chemotherapy in this group of patients. When we compared the use of chemotherapy with SCRS to no chemotherapy, the median survival was 12.0 years in both groups and, of course, no significance for this treatment was apparent. Also, a change in the HIPEC between index CRS and SCRS did not bring about a difference in survival. The suggestion made by Brouquet et al^21^ that the chemotherapy regimen between CRS and SCRS must be changed could not be validated.^21^ Our data shows that hyperthermic perioperative chemotherapy did not improve survival as compared with no perioperative chemotherapy or EPIC. Also, a comparison of HIPEC mitomycin C, HIPEC cisplatin and doxorubicin, HIPEC melphalan, and HIPEC mitomycin C and doxorubicin showed no differences in the type of HIPEC used. In the multivariant analysis in Table 4, a HIPEC treatment was always superior to no perioperative chemotherapy or EPIC. Perhaps a reasonable conclusion from these data is that HIPEC does improve survival but selection of the best HIPEC from these nonrandomized data was not possible.

### Limitations

There are limitations to this study that must be considered before these data can be applied clinically. These data come from a single institution and may not be relevant at other peritoneal surface malignancy treatment centers. The data was accumulated over 3 decades. A single HIPEC methodology, an open technique with manual heat and chemotherapy distribution, was used.^31^ The relevance of these data to centers using the closed HIPEC method is not known. Of great interest would be a histopathologic and/or molecular assessment of the clinical material obtained at the time of CRS and a direct comparison looking for more aggressive disease at the time of SCRS. Histopathologic comparisons for this evaluation were not performed prospectively and are unavailable. Also, 4 different chemotherapy agents as HIPEC were used. Clinical judgments rather than protocol were used to select the chemotherapy agent within the hyperthermic chemotherapy solution used to lavage the abdomen and pelvis. Perhaps the major limitation, in the long run, to all work with peritoneal metastases and regional chemotherapy is its interdependence upon immunologic, molecular, and systemic chemotherapy advances with these diseases. The optimal combination of systemic and local-regional treatments has not yet been defined.

## References

[1] Carr NJ Cecil TD Mohamed F; Peritoneal Surface Oncology Group International. A consensus for classification and pathologic reporting of pseudomyxoma peritonei and associated appendiceal neoplasia: the results of the Peritoneal Surface Oncology Group International (PSOGI) modified delphi process. Am J Surg Pathol. 2016;40:14–26. 26492181 10.1097/PAS.0000000000000535

[2] Long RTL Spratt JS Jr Dowling E. Pseudomyxoma peritonei: new concepts in management with a report of seventeen patients. Am J Surg. 1969;117:162–169. 5773930 10.1016/0002-9610(69)90300-6

[3] Sugarbaker PH. Epithelial appendiceal neoplasms. Cancer J. 2009;15:225–235. 19556909 10.1097/PPO.0b013e3181a9c781

[4] Sugarbaker PH. New standard of care for appendiceal epithelial malignancies and pseudomyxoma peritonei syndrome. Lancet Oncol. 2006;7:69–76. 16389186 10.1016/S1470-2045(05)70539-8

[5] Nagarajan P Renehan A Saunders MP. Sugarbaker procedure for pseudomyxoma peritonei. Cochrane Database of Systematic Reviews 2006. 1. CD005659.

[6] Bryant J Clegg AJ Sidhu MK. Systematic review of the Sugarbaker procedure for pseudomyxoma peritonei. Br J Surg. 2005;92:153–158. 15685704 10.1002/bjs.4862

[7] Baumgartner JM Srivastava A Melnitchouk N. A multi-institutional study of peritoneal recurrence following resection of low-grade appendiceal mucinous neoplasms. Ann Surg Oncol. 2021;28:4685–4694. 33415564 10.1245/s10434-020-09499-y

[8] Esquivel J Sugarbaker PH. Elective surgery in recurrent colon cancer with peritoneal seeding: when to and when not to proceed. (Editorial) Cancer Ther. 1998;1:321–325.

[9] Mohamed F Chang D Sugarbaker PH. Third look surgery and beyond for appendiceal malignancy with peritoneal dissemination. J Surg Oncol. 2003;83:5–12; discussion 12. 12722090 10.1002/jso.10234

[10] Jacquet P Sugarbaker PH. Current methodologies for clinical assessment of patients with peritoneal carcinomatosis. J Exp Clin Cancer Res. 1996;15:49–58. 10.1007/978-1-4613-1247-5_23 8849962

[11] Van der Speeten K Stuart OA Sugarbaker PH. Cancer chemotherapy for peritoneal metastases. Pharmacology and treatment. In Sugarbaker PH (Ed). Cytoreductive Surgery & Perioperative Chemotherapy for Peritoneal Surface Malignancy. Textbook and Video Atlas. 2nd Edition. Woodbury, CT: Cine-Med Publishing:2017. 47–82.

[12] Ronnett BM Zahn CM Kurman RJ. Disseminated peritoneal adenomucinosis and peritoneal mucinous carcinomatosis: a clinicopathologic analysis of 109 cases with emphasis on distinguishing pathologic features, site of origin, prognosis, and relationship to “pseudomyxoma peritonei.”. Am J Surg Pathol. 1995;19:1390–1408. 7503361 10.1097/00000478-199512000-00006

[13] Misdraji J Yantiss RK Graeme-Cook FM. Appendiceal mucinous neoplasms. a clinicopathologic analysis of 107 cases. Am J Surg Pathol. 2003;27:1089–1103. 12883241 10.1097/00000478-200308000-00006

[14] Valasek MA Pai RK. An update on the diagnosis, grading, and staging of appendiceal mucinous neoplasms. Adv Anat Pathol. 2018;25:38–60. 29016471 10.1097/PAP.0000000000000178

[15] Sugarbaker PH Chang D Liang J. Similar survival of all subtypes of mucinous appendiceal adenocarcinoma except the intermediate subtype which shows improved survival. Ann Surg Oncol. 2023;30:1874–1885. 36542246 10.1245/s10434-022-12864-8

[16] Sugarbaker PH Chang D. Lymph node positive pseudomyxoma peritonei. Eur J Surg Oncol. 2022;48:2369–2377. 35941031 10.1016/j.ejso.2022.07.018

[17] Esquivel J Sugarbaker PH. Second-look surgery in patients with peritoneal dissemination of appendiceal malignancy. analysis of prognostic factors in 98 patients. Ann Surg. 2001;234:198–205. 11505065 10.1097/00000658-200108000-00009 PMC1422006

[18] Sugarbaker PH Alderman R Edwards G. Prospective morbidity and mortality assessment of cytoreductive surgery plus perioperative intraperitoneal chemotherapy to treat peritoneal dissemination of appendiceal mucinous malignancy. Ann Surg Oncol. 2006;13:635–644. 16523363 10.1245/ASO.2006.03.079

[19] Sugarbaker PH van der Speeten K Stuart OA Chang D Mahteme H. Patient- and treatment-related variables, adverse events and their statistical relationship for treatment of peritoneal metastases. In Sugarbaker PH (Ed). Cytoreductive Surgery & Perioperative Chemotherapy for Peritoneal Surface Malignancy. Textbook and Video Atlas. 2nd Edition. Woodbury, CT: Cine-Med Publishing. 2017:207–230.

[20] Yan TD Bijelic L Sugarbaker PH. Critical analysis of treatment failure after complete cytoreductive surgery and perioperative intraperitoneal chemotherapy for peritoneal dissemination from appendiceal mucinous neoplasms. Ann Surg Oncol. 2007;14:2289–2299. 17541772 10.1245/s10434-007-9462-0

[21] Brouquet A Goéré D Lefèvre JH. The second procedure combining complete cytoreductive surgery and intraperitoneal chemotherapy for isolated peritoneal recurrence: postoperative course and long-term outcome. Ann Surg Oncol. 2009;16:2744–2751. 19626375 10.1245/s10434-009-0611-5

[22] Sardi A Jimenez WA Nieroda C. Repeated cytoreductive surgery and hyperthermic intraperitoneal chemotherapy in peritoneal carcinomatosis from appendiceal cancer: analysis of survival outcomes. Eur J Surg Oncol. 2013;39:1207–1213. 24007834 10.1016/j.ejso.2013.08.017

[23] Ahmadi N Kostadinov D Sakata S. Managing recurrent pseudomyxoma peritonei in 430 patients after complete cytoreduction and HIPEC: a dilemma for patients and surgeons. Ann Surg Oncol. 2021;28:7809–7820. 34041626 10.1245/s10434-021-10093-z

[24] Sugarbaker PH Chang D. Secondary cytoreductive surgery for lymph node positive mucinous appendiceal neoplasms. Surg Oncol. 2023;46:101903. 36652898 10.1016/j.suronc.2023.101903

[25] Votanopoulos KI Newman NA Russell G. Outcomes of cytoreductive surgery (CRS) with hyperthermic intraperitoneal chemotherapy (HIPEC) in patients older than 70 years; survival benefit at considerable morbidity and mortality. Ann Surg Oncol. 2013;20:3497–3503. 23780382 10.1245/s10434-013-3053-z PMC3881978

[26] Sugarbaker PH. After thirty years of experience with early postoperative intraperitoneal 5-fluorouracil now saying goodbye. Surg Oncol. 2022;42:101757. 35398760 10.1016/j.suronc.2022.101757

[27] Jacquet P Sugarbaker PH. Abdominal adhesions causing intestinal obstruction following cytoreductive surgery and early postoperative intraperitoneal chemotherapy. Acta Chir Austriaca. 1995;27:92–95.

[28] Klausner JM Lelcuk S Inbar M. The effects of perioperative fluorouracil administration on convalescence and wound healing. Arch Surg. 1986;121:239–242. 3947222 10.1001/archsurg.1986.01400020125017

[29] Sugarbaker PH Chang D. Secondary cytoreductive surgery for low-grade appendiceal mucinous neoplasms. J Surg Oncol. 2022;126:1451–1461. 35975822 10.1002/jso.27064

[30] Sugarbaker PH Chang D. Secondary cytoreductive surgery for 86 patients with mucinous appendiceal adenocarcinoma. J Surg Oncol. 2023;127:999–1010. 36734844 10.1002/jso.27208

[31] Sugarbaker PH Averbach AM Jacquet P Stephens AD Stuart OA. A simplified approach to hyperthermic intraoperative intraperitoneal chemotherapy (HIIC) using a self retaining retractor. In Sugarbaker PH (ed); Peritoneal Carcinomatosis: Principles of Management. Boston: Kluwer. 1996:415–421. 10.1007/978-1-4613-1247-5_26 8849965

